# Regulation of rod photoreceptor function by farnesylated G-protein γ-subunits

**DOI:** 10.1371/journal.pone.0272506

**Published:** 2022-08-08

**Authors:** Alexander V. Kolesnikov, Elena Lobysheva, Jaya P. Gnana-Prakasam, Vladimir J. Kefalov, Oleg G. Kisselev

**Affiliations:** 1 Department of Ophthalmology, Gavin Herbert Eye Institute, University of California, Irvine, CA, United States of America; 2 Department of Ophthalmology and Visual Sciences, Washington University School of Medicine, St. Louis, Missouri, United States of America; 3 Department of Ophthalmology, Saint Louis University School of Medicine, Saint Louis, Missouri, United States of America; 4 Department of Biochemistry and Molecular Biology, Saint Louis University School of Medicine, Saint Louis, Missouri, United States of America; 5 Department of Physiology and Biophysics, University of California, Irvine, CA, United States of America; Doheny Eye Institute/UCLA, UNITED STATES

## Abstract

Heterotrimeric G-protein transducin, Gt, is a key signal transducer and amplifier in retinal rod and cone photoreceptor cells. Despite similar subunit composition, close amino acid identity, and identical posttranslational farnesylation of their Gγ subunits, rods and cones rely on unique Gγ_1_ (*Gngt1*) and Gγ_c_ (*Gngt2*) isoforms, respectively. The only other farnesylated G-protein γ-subunit, Gγ_11_ (*Gng11*), is expressed in multiple tissues but not retina. To determine whether Gγ_1_ regulates uniquely rod phototransduction, we generated transgenic rods expressing Gγ_1_, Gγ_c_, or Gγ_11_ in Gγ_1_-deficient mice and analyzed their properties. Immunohistochemistry and Western blotting demonstrated the robust expression of each transgenic Gγ in rod cells and restoration of Gα_t1_ expression, which is greatly reduced in Gγ_1_-deficient rods. Electroretinography showed restoration of visual function in all three transgenic Gγ_1_-deficient lines. Recordings from individual transgenic rods showed that photosensitivity impaired in Gγ_1_-deficient rods was also fully restored. In all dark-adapted transgenic lines, Gα_t1_ was targeted to the outer segments, reversing its diffuse localization found in Gγ_1_-deficient rods. Bright illumination triggered Gα_t1_ translocation from the rod outer to inner segments in all three transgenic strains. However, Gα_t1_ translocation in Gγ_11_ transgenic mice occurred at significantly dimmer background light. Consistent with this, transretinal ERG recordings revealed gradual response recovery in moderate background illumination in Gγ_11_ transgenic mice but not in Gγ_1_ controls. Thus, while farnesylated Gγ subunits are functionally active and largely interchangeable in supporting rod phototransduction, replacement of retina-specific Gγ isoforms by the ubiquitous Gγ_11_ affects the ability of rods to adapt to background light.

## Introduction

The high sensitivity of rod photoreceptors is achieved by the activation of multiple copies of the heterotrimeric G-protein, Gt, by a single rhodopsin [1]. The Gtβγ (Gβ_1_γ_1_) complex is crucial for efficient signal amplification in mouse rods. Analysis of Gγ_1_-deficient rods has shown that although Gα_t1_ is sufficient for signal transduction, the efficient signal amplification required for nocturnal vision is achieved only in the presence of the Gtβγ-complex [2, 3]. Whether the isoform diversity among Gγ-subunits contributes to specific physiological characteristics of retinal photoreceptors remains unknown. For example, rod and cone Gt heterotrimers are considered unique and the sole signal transducers in rods and cones respectively, compared to other cell types that contain multiple G-protein isoforms. Replacing individual subunits in retinal photoreceptors is a powerful approach to address their functional differences. Each of the three subunits of transducin, rod Gα_t1_ vs. cone Gα_t2_, rod Gβ_1_ vs. cone Gβ_3_, and rod Gγ_1_ vs. cone Gγ_c_, can potentially contribute to the observed lower rate of Gt activation in cones. With rare exception [4], the majority of the data obtained from Gα_t1_ replacement experiments point to close functional similarity and good interchangeability between Gα_t1_ and Gα_t2_ [5–7]. Thus, the lower visual sensitivity of cones compared to rods and reduced rate of signal transduction between the cone visual pigment and PDE cannot be explained by the differences in the Gtα subunits.

**Fig 1:**
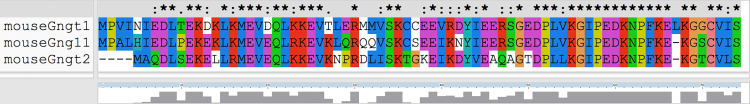
Multiple amino acid sequence alignment of mouse rod Gγ_1_ (*Gngt1*), cone Gγ_c_ (*Gngt2*), and Gγ_11_ (*Gng11*).

G-protein γ-subunits are a protein family composed of twelve isoforms that are posttranslationally isoprenylated and carboxymethylated [8–11]. Only three Gγ subunits are modified by a 15-carbon farnesyl, while the rest contain a 20-carbon geranylgeranyl lipid moiety. The three farnesylated Gγ subunits are: rod-specific Gtγ_1_ (Gγ_1_, *Gngt1*) [12]; cone-specific Gtγ_c_ (Gγ_c_, Gγ_9_, *Gngt2*) [13]; and the relatively ubiquitous Gγ_11_ (*Gng11*) [14]. Rod and cone subunits of transducin share fairly high levels of amino acid identity: Gα_t1_ is 78% identical to Gα_t2_, Gβ_1_ is 80% identical to Gβ_3_, while Gγ_1_ is 64% identical to Gγ_c_ (Fig 1). Despite their similarities, Gγ subunits differ dramatically in their tissue expression pattern and putative G-protein coupled receptor (GPCR) partners [15, 16]. The reason for this intriguing diversity of Gγ subunits and the contribution of their amino acid sequence and protein structure in G-protein signaling remain very poorly understood. Thus, it is still a mystery why Gγ_1_ is specifically expressed in the rod photoreceptors and Gγ_c_ is exclusive to the cones, while Gγ_11_ is excluded from both photoreceptor types.

The determination of physiological roles of Gγ subunits in non-photoreceptor cells is difficult due to the redundancy of G-protein mediated pathways [17]. Phototransduction in rods, however, is mediated by a single G-protein transducin, Gtαβγ (Gα_t1_, Gβ_1_, Gγ_1_). Deletion of *Gngt1* to generate Gγ_1_-deficient mice results in rods with greatly reduced signal amplification and is associated with severe reduction in the expression of Gα_t1_ and Gβ_1_ [2]. To address how the specific properties of Gγ regulate the function of rods, we created transgenic mice expressing the rod Gγ_1_, the cone Gγ_c_, or the ubiquitous Gγ_11_ in the *Gngt1*^*-/-*^ line. This approach allowed us to determine whether substitution of Gγ_1_ by Gγ_c_ or Gγ_11_ restores rod function. We also analyzed how the expression of each Gγ affects the expression of Gα_t1_ and Gβ_1_, as well as their light-driven translocation within rods.

## Materials and methods

### Generation of Gγ transgenic mouse lines

All experiments were performed in accordance with the Guide for the Care and Use of Laboratory Animals and were approved by the Saint Louis University Institutional Animal Care and Use Committee and the Washington University Animal Studies Committee. Unless otherwise specified, all mice were age-matched 2- to 3-month-old littermates of either sex; they were kept under the standard 12 h dark/light cycle and dark-adapted overnight before all experiments.

**Fig 2:**
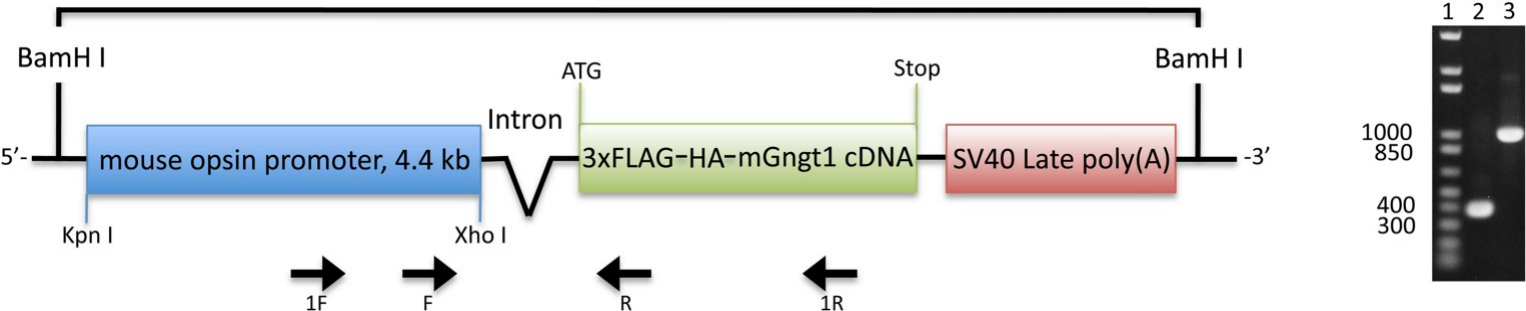
Gγ_1_ transgenic construct (left) and the PCR screening test (right). DNA gel: 1) Molecular weight markers; 2) 349 bp PCR product using universal F/R primers; 3) 1011 bp PCR product using 1F/1R primers. Similar design was employed to generate Gγ_c_ and Gγ_11_ transgenic constructs.

We introduced three individual mouse Gγ-subunits into Gγ_1_-deficient rods [18]. All transgenic constructs included the 4.4 kb mouse opsin promoter (generous gift from Dr. Lem, Tufts Medical Center) [19], mouse Gngt1 cDNA, as well as appropriate intron and poly(A) sequences (Fig 2). An in-frame insertion of 3xFLAG-HA epitope at the N-terminus of all Gγ was designed to help with detection and quantification of the expressed proteins. The following nucleic acid sequence was present in all individual synthetic genes used to generate the three transgenic constructs: tttaaactgcagaagttggtcgtgaggcactgggcaggtaagtatcaaggttacaagacaggtttaaggagaccaatagaaactgggcttgtcgagacagagaagactcttgcgtttctgataggcacctattggtcttactgacatccactttgcctttctctccacaggtgtccactcccagttcaattacagctcttaaggctagagtacttaatacgactcactataggctagcctcgatcgagaattcacgcgtcttccctgacagaagatggactacaaagaccatgacggtgattataaagatcatgacatcgattacaaggatgacgatgacaagcttgcggccgcgaattcatacccatacgacgtaccagattacgct.

It included part of the intron and 3xFLAG-HA epitope, and was used for developing genotyping assay at Transnetyx, Inc. The genotyping strategy is available for sharing upon request. The purified *BamH1* insertion fragment was microinjected into fertilized mouse eggs and re-implanted in pseudopregnant C57Bl/6 female mice. Founders expressing Gγ_1_, Gγ_c_, and Gγ_11_ transgenes were bred with our existing Gγ_1_-deficient line, *Gngt1*^*-/-*^, to generate *Gγ*_*1*_^*+*^*Gngt1*^*-/-*^, *Gγ*_*c*_^*+*^*Gngt1*^*-/-*^, and *Gγ*_*11*_^*+*^*Gngt1*^*-/-*^ mice.

### Western blotting and antibodies

Retinas from 2-month-old dark-adapted mice were dissected, flash-frozen in liquid nitrogen, and stored at -80°C until protein quantification or biochemical experiments. Bio-Rad precast 12% Mini-Protean TGX were used for all SDS-gels. Protein transfer was performed using Trans-Blot SD semi-dry cell on PVDF membrane. Rabbit antibodies sc-389-Gα_t1_, sc-15382-rhodopsin were from Santa Cruz Biotechnology (Santa Cruz, CA). Mouse FLAG M2 F1804 were from Sigma-Aldrich. Rabbit HA TA150084 were from Origene. Rabbit PDE6A PA1-720, PDE6G PA1-723 and beta Actin PA1-16889 and secondary HRP antibodies were from Invitrogen. Rabbit antibodies against Gβ_1_ and Gγ_1_ were a gift from N. Gautam (Washington University, St. Louis, MO). Primary antibody dilution was 1:1,000. Secondary antibody dilution was 1:10,000. All gels/blots were developed and analyzed in compliance with the digital image and integrity policies. Prior to blocking non-specific binding by 5% BSA in TBST, the PVDF membranes were cut to size using Amersham Rainbow molecular weight markers as a guide. For proteins with significantly different molecular weights, such as Gα_t1_ and Gγ_1_, the membrane was cut in half horizontally into the upper and lower portions, which were stained with individual antibodies. After staining with primary and secondary antibodies, blots were developed using Amersham ECL Prime detection kit. Chemiluminescence was visualized using Li-COR C-DiGit^®^ Blot Scanner that was setup to collect and save time-lapse data in the high-sensitivity mode. Quantitation was performed using Image Studio software. The pixel saturation tool was used to ensure that optical density (OD) of protein bands is not saturated, and only unsaturated bands in a linear range of protein band intensities were used for quantitation. Local background was subtracted.

### Light microscopy and immunohistochemistry

For immune labeling, eyes were cryo-preserved in Tissue-Tek O.C.T. compound. Semi-thin 0.9-μm sections were cut in the dorsal-to-ventral direction through the optic nerve and immunostained as previously described [20]. Images were taken on a Leica DM 5500 D microscope using DFC360 FX camera.

For the Gα_t1_ translocation experiment, mice were dark-adapted overnight, their eyes were dilated with one drop of 1% atropine sulfate and then exposed for 15 minutes to steady white background light of various intensities, measured by Sper Scientific Advanced Light Meter 840022, followed by euthanasia by CO_2_ and eye cryo-preservation. Unsaturated pictures of cross-sections of the retina immunolabelled with anti-Gα_t1_ antibody were analyzed in Adobe Photoshop CS4 Extended using the analysis module. Integrated density (ID) was measured in the rod outer segment (OS), and combined area of rod inner segment (IS), rod outer nuclear layer (ONL) and outer plexiform layer (OPL) in three independent sections. ID_OS_+(ID_IS_+OD_ONL_+OD_OPL_) was taken as 100% followed by the calculation of the proportion of Gα_t1_ in OS as ID_OS_ in percent.

### *In vivo* electroretinography (ERG)

Animals were dark-adapted overnight and anesthetized by subcutaneous injection of ketamine (80 mg/kg) and xylazine (15 mg/kg). Pupils were dilated with 1% atropine sulfate. During testing, a heating pad controlled by a rectal temperature probe maintained body temperature at 37–38°C. Full-field ERGs were recorded using a UTAS BigShot apparatus (LKC Technologies) and corneal cup electrodes, as described [21]. The reference electrode needle was inserted under the skin at the skull. Test flashes of white light ranging from 2.5x10^-5^ cd∙s m^-2^ to 700 cd∙s m^-2^ were applied in darkness (scotopic conditions). Responses from several trials were averaged and the intervals between trials were adjusted so that responses did not decrease in amplitude over the series of trials for each step. The recorded responses were low-pass filtered at 500 Hz.

### Single-cell suction recordings

Mice were dark-adapted overnight, sacrificed by CO_2_ asphyxiation, and their retinas were removed under infrared illumination. Retinas were chopped into small pieces with a razor blade and transferred to a perfusion chamber on the stage of an inverted microscope. A single rod outer segment on the edge of a retina piece was drawn into a glass microelectrode filled with solution containing 140 mM NaCl, 3.6 mM KCl, 2.4 mM MgCl_2_, 1.2 mM CaCl_2_, 3 mM HEPES (pH 7.4), 0.02 mM EDTA, and 10 mM glucose. The perfusion solution contained 112.5 mM NaCl, 3.6 mM KCl, 2.4 mM MgCl_2_, 1.2 mM CaCl_2_, 10 mM HEPES (pH 7.4), 20 mM NaHCO_3_, 3 mM Na succinate, 0.5 mM Na glutamate, 0.02 mM EDTA, and 10 mM glucose. The solution was bubbled with 95% O_2_ / 5% CO_2_ mixture and its temperature was maintained at 37°C with an in-line ceramic heater.

Rods were stimulated with 20-ms test flashes of calibrated 500 nm light. The light intensity was controlled with neutral density filters in 0.5 log unit steps. Photoresponses were amplified, low-pass filtered (30 Hz, 8-pole Bessel), and digitized (1 kHz). Data were analyzed using Clampfit 10.6 and Origin 8.5 software. Intensity-response relationships were fitted with Naka-Rushton hyperbolic function:

$$R=\frac{{R_{max}∙I}^{n}}{I^{n}+I_{1/2}^{n}},$$ (1)

where *R* is the transient-peak amplitude of the rod response, *R*_max_ is the maximal response amplitude, *I* is the flash intensity, *n* is the Hill coefficient (exponent), and *I*_1/2_ is the half-saturating light intensity. Normalized rod flash sensitivity (*S*_*f*_) was calculated from the linear part of the intensity-response curve, as follows:

$$S_{f}=\frac{R}{R_{max}∙I},$$ (2)

where *R* is the amplitude of dim flash response, *R*_*max*_ is the maximal response amplitude for that cell, and *I* is the flash strength used to elicit the dim flash response.

The amplification of the rod phototransduction cascade was evaluated from test flash intensities that produced identical rising phases of dim flash responses. This approach was preferred to calculation of the amplification constant by the method of Lamb and Pugh [22], due to the relatively long duration of test flashes and the effect of low-pass filtering on the response front. Integration time (*T*_integr._) was calculated as the integral of the dim flash response with the transient peak amplitude normalized to unity. The time constant of the dim flash response recovery (*τ*_rec_) was derived from single-exponential fit to the falling phase of the response. The dominant recovery time constant (*τ*_D_) was determined from supersaturating flashes [23], using a 10% criterion for recovery of the photocurrent from saturation.

### Transretinal ERG recordings

Mice were dark-adapted overnight and sacrificed by CO_2_ asphyxiation. The whole retina was removed from each mouse eyecup under infrared illumination and stored in oxygenated aqueous L15 (13.6 mg/ml, pH 7.4) solution (Sigma-Aldrich) containing 0.1% BSA, at RT. The retina was mounted on filter paper with the photoreceptor side up and placed in a perfusion chamber [24] between two electrodes connected to a differential amplifier. The tissue was perfused with bicarbonate-buffered Locke’s solution supplemented with 2 mM L-glutamate and 10 μM DL-2-amino-4-phosphonobutyric acid to block postsynaptic components of the photoresponse [25], and with 20 μM BaCl_2_ to suppress the slow glial PIII component [26]. The perfusion solution was continuously bubbled with a 95% O_2_ / 5% CO_2_ mixture and heated to 36–37°C.

The photoreceptors in the retina were stimulated with 20-ms test flashes of calibrated 505 nm LED light. The light intensity was controlled by a computer in 0.5 log unit steps. The prolonged (> 1 h) background illumination was achieved with the same 505 nm LED activating ~830 rhodopsin molecules (R*) per rod per second initially. Photoresponses were amplified by a differential amplifier (DP-311, Warner Instruments), low-pass filtered at 30 Hz (8-pole Bessel), and digitized at 1 kHz. Data were analyzed with Clampfit 10.6 and Origin 8.5 software.

### Statistical analysis

For all experiments, data were expressed as mean ± SEM and analyzed with the independent two-tailed Student’s *t*-test (using an accepted significance level of *p* < 0.05).

## Results

### Generation of the three transgenic Gγ lines

The transgenic mice were generated using the construct shown in Fig 2. We used the mouse opsin promoter to target the expression of each of the three transgenic Gγ subunits selectively in rod photoreceptors. We also included a 3xFLAG and an HA tag to facilitate detection of the transgenic protein in the retina. Upon the successful generation of the three Gγ_1_, Gγ_c_, and Gγ_11_ transgenic strains, we crossed them with the rod Gγ_1_-deficient (*Gngt1*^*-/-*^) line to effectively substitute the rod Gγ_1_ with each of the transgenic Gγ subunits. As we have shown previously, deletion of rod Gγ_1_ in mice results in dramatic suppression of rod sensitivity and reduction in the expression of the other two rod transducin subunits, Gα_t1_ and Gβ_1_ [2], see also [3]. Thus, generating *Gγ*_*1*_^*+*^*Gngt1*^*-/-*^, *Gγ*_*c*_^*+*^*Gngt1*^*-/-*^, and *Gγ*_*11*_^*+*^*Gngt1*^*-/-*^ mice allowed us to investigate how the substitution of the endogenous rod Gγ_1_ subunit with transgenic Gγ_1_ (as a control), or with Gγ_c_ or Gγ_11_ will affect the Gt expression profile and functional properties of mouse rods.

**Fig 3:**
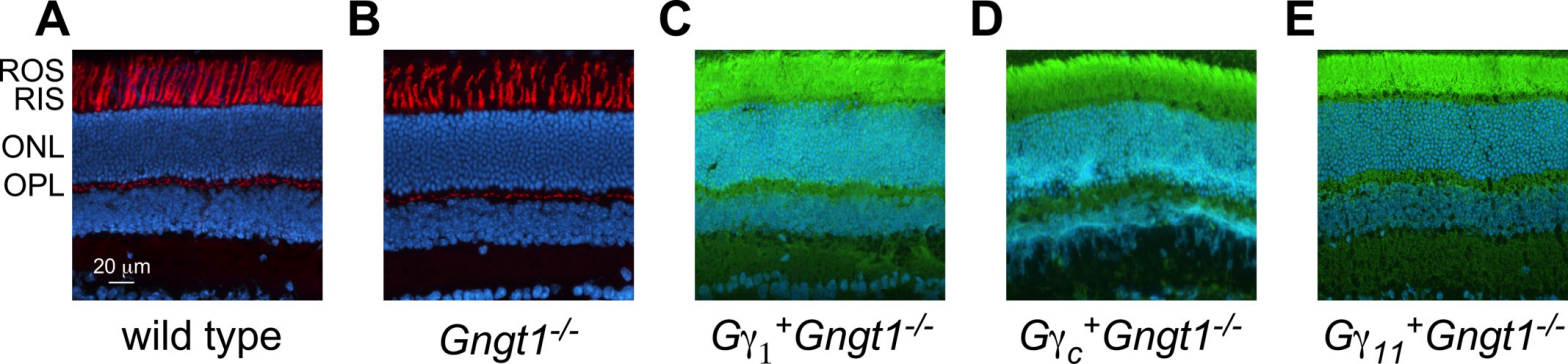
Immunohistochemical analysis of the transgenic protein expression using anti-FLAG antibodies (green), with DAPI counterstaining (blue), at P30. (**A**) and (**B**) are also counterstained with wheat germ agglutinin (red) to highlight ROS/RIS. Cryo-sections, 40x. (**A**) wild type, (**B**) *Gngt1*^*-/-*^, (**C**) *Gγ*_*1*_^*+*^*Gngt1*^*-/-*^, (**D**) *Gγ*_*c*_^*+*^*Gngt1*^*-/-*^, (**E**) *Gγ*_*11*_^*+*^*Gngt1*^*-/-*^ retinas. ROS–rod outer segments, RIS–rod inner segments, ONL–outer nuclear layer, OPL–outer plexiform layer.

We began our analysis by investigating the expression localization of the Gγ_1_, Gγ_c_, and Gγ_11_ γ-subunits in their respective transgenic mouse retinas. To prevent light-driven translocation and ensure that all Gt subunits were properly localized in the outer segments of rods, these experiments were performed after dark-adapting the animals overnight. Using an anti-FLAG antibody staining of retinal sections, we found, as expected, that no transgenic protein was found in wild type or *Gngt1*^*-/-*^ retinas (Fig 3A and 3B). Transgenic Gγ_1_, Gγ_c_, and Gγ_11_ subunits were all, indeed, localized in the outer segments of rods (Fig 3C–3E). Thus, in addition to the transgenically reintroduced Gγ_1_, both cone Gγ_c_ and the non-photoreceptor Gγ_11_ were targeted properly to the rod outer segments following dark adaptation.

The level of transducin in rod outer segments is directly proportional to the amplification of rod phototransduction [27], making its proper translocation crucial for the function of rods. Our finding that all three transgenic Gγ subunits localized properly to the rod outer segments was critical for enabling us to perform the subsequent physiological analysis of the three transgenic mouse lines and to compare directly their functional properties. Notably, our immunohistochemical analysis also showed that all three transgenic lines retained normal retina morphology and uniform expression of the transgenic proteins in the Gγ_1_-deficient rods.

### Restoration of transducin complement in all Gγ-expressing lines

**Fig 4:**
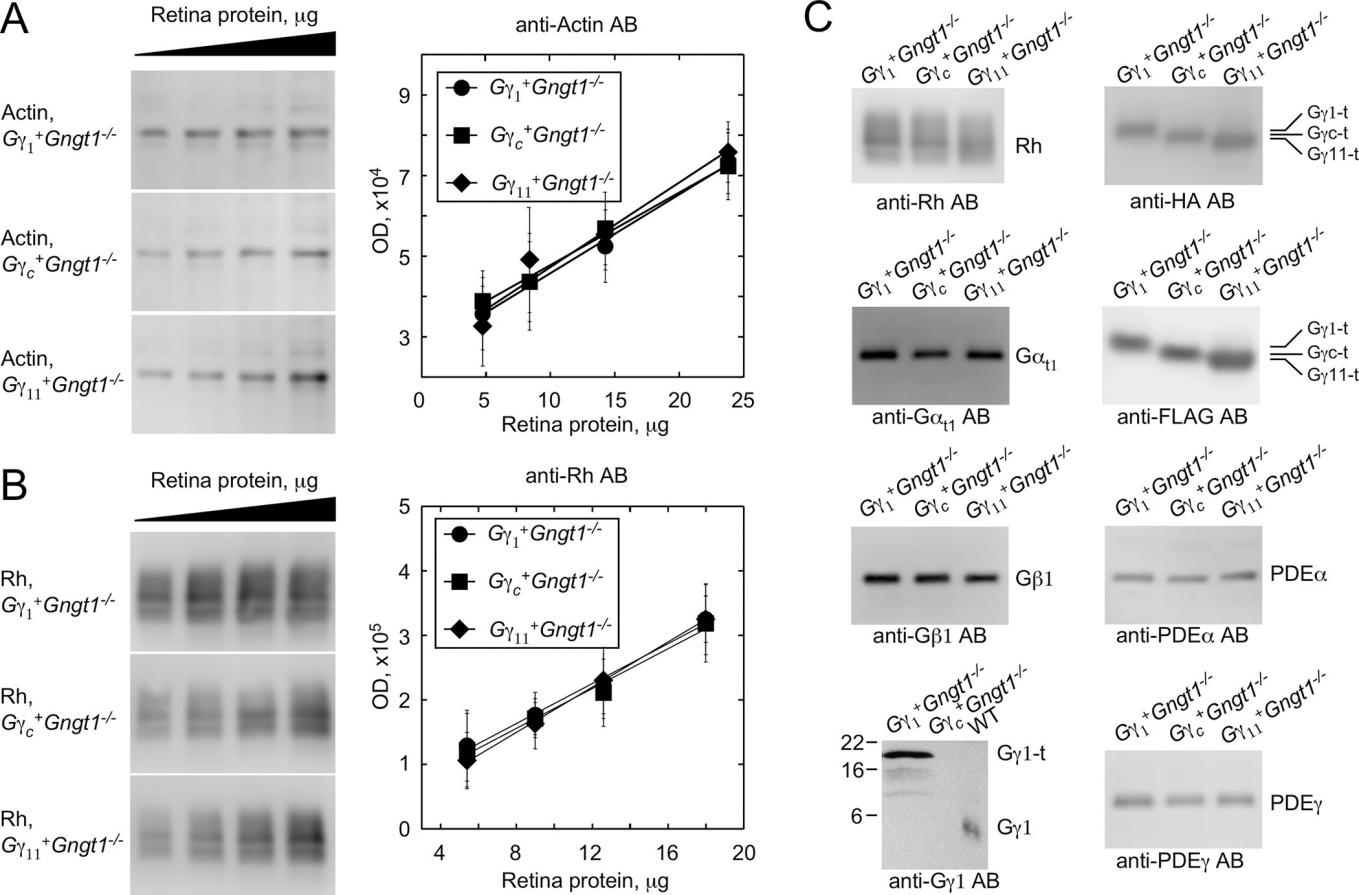
Western blot analysis of retina homogenates obtained from indicated transgenic mice. Representative staining for actin (**A**) and rhodopsin (**B**) in samples with progressively increasing amounts of loaded retina homogenate obtained from *Gγ*_*1*_^*+*^*Gngt1*^*-/-*^, *Gγ*_*c*_^*+*^*Gngt1*^*-/-*^, and *Gγ*_*11*_^*+*^*Gngt1*^*-/-*^ mice. Graph shows optical density of Western blot bands against amount of total retina protein (n = 3). Linearity of plots demonstrates sub-saturating ECL signal ensuring direct quantitative comparison. (**C**) Comparative staining of samples from the *Gγ*_*1*_^*+*^*Gngt1*^*-/-*^, *Gγ*_*c*_^*+*^*Gngt1*^*-/-*^, and *Gγ*_*11*_^*+*^*Gngt1*^*-/-*^ retina homogenates using indicated antibodies against rhodopsin, Gα_t1_, Gβ_1,_ Gγ_1,_ HA, FLAG, PDEα, and PDEγ subunits.

Quantitative Western blot analysis was performed in the linear portion of the dose escalation plots of the total retina protein vs. optical densities of the protein bands to assure the Western signal is not saturated, typically in the 5–20 μg range. It showed that expression levels of general cellular protein actin and rhodopsin in the retina were comparable in *Gγ*_*1*_^*+*^*Gngt1*^*-/-*^, *Gγ*_*c*_^*+*^*Gngt1*^*-/-*^, and *Gγ*_*11*_^*+*^*Gngt1*^*-/-*^ mice (Fig 4A and 4B), a finding consistent with the normal morphology and lack of degeneration in these retinas (Fig 3). Direct protein expression comparison in Fig 4C used 10 μg of retina protein in each sample. Gγ_1_, Gγ_c_, and Gγ_11_ transgenic proteins were easily identified by both anti-FLAG and anti-HA staining (Fig 4C). Expression levels of the three γ-subunits also appeared similar by this test. Gγ_1_-specific antibodies stained transgenic Gγ_1_ stronger, compared to the native Gγ_1_ in WT samples (Fig 4C, bottom), which may be explained either by higher level of transgenic protein whose expression is driven by the strong *rhodopsin* promoter compared to the *Gngt1* promoter in wild type retinas, or possibly by better accessibility of the N-terminal epitope in the transgenic protein. Western blots also showed that expression of each of the transgenic Gγ subunits restores the amounts of Gα_t1_ to wild type levels (Fig 4C). Restoration of Gα_t1_ expression in all transgenic lines was also corroborated by the robust staining and proper Gα_t1_ localization to the rod outer segments in dark adapted retinas, discussed separately in Fig 8. The expression levels of Gβ_1_ were also recovered (Fig 4C). As expected, all three transgenic retinas expressed equal amounts of the effector protein PDE6, as judged by the similar intensities of protein bands for PDE6α and PDE6γ (Fig 4C). Thus, transgenic retinas appeared to express the full and equal sets of rhodopsin, transducin, and PDE.

### Restoration of scotopic visual function in all Gγ-expressing lines

**Fig 5:**
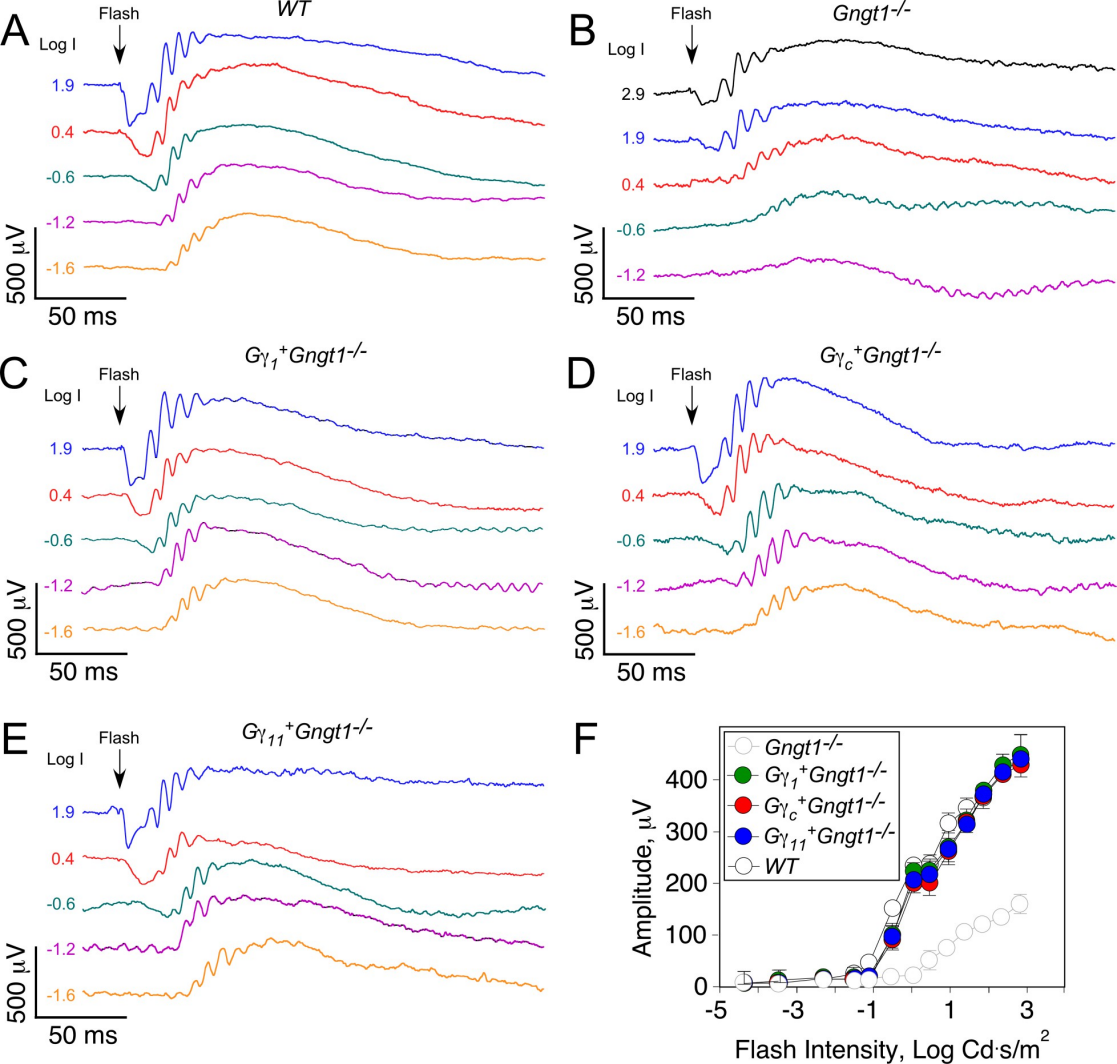
Families of *in vivo* ERG responses from wild type **(A)**, *Gngt1*^-/-^ **(B)**, *Gγ*_*1*_^*+*^*Gngt1*^*-/-*^ **(C)**, *Gγ*_*c*_^*+*^*Gngt1*^*-/-*^ **(D)**, and *Gγ*_*11*_^*+*^*Gngt1*^*-/-*^ **(E)** mice. Waveforms are color coded according to the white flash of indicated intensity. (**E**) Averaged scotopic *in vivo* ERG intensity-response functions (mean ± SEM) for wild type (n = 3), *Gngt1*^-/-^ (n = 3), *Gγ*_*1*_^*+*^*Gngt1*^*-/-*^ (n = 3), *Gγ*_*c*_^*+*^*Gngt1*^*-/-*^ (n = 3), and *Gγ*_*11*_^*+*^*Gngt1*^*-/-*^ (n = 3) mouse lines.

To determine how the expression of each of the three Gγ-subunits affects the functional properties of rods, we first performed electroretinography (ERG) analysis of control wild type and *Gngt1*^*-/-*^ mice and the transgenic *Gγ*_*1*_^*+*^*Gngt1*^*-/-*^, *Gγ*_*c*_^*+*^*Gngt1*^*-/-*^, and *Gγ*_*11*_^*+*^*Gngt1*^*-/-*^ mice *in vivo* (Fig 5A–5E). As we have previously shown [2], deletion of the rod Gγ_1_-subunit results in substantial desensitization and reduction in the maximal ERG a-wave response (Fig 5F, open light grey circles). Notably, expression of Gγ_1_, Gγ_c_, or Gγ_11_ in the *Gngt1*^*-/-*^ mice (Fig 5F, filled circles) all restored robust scotopic function essentially to the wild type level (Fig 5F, open black circles; see also [28] for the reference to wild type data). Thus, not only did the transgenic expression of Gγ_1_ rescue scotopic vision in the Gγ_1_-deficient mice, but the same effect could be achieved by expressing the cone Gγ_c_ or the non-photoreceptor Gγ_11_.

### Restoration of rod photosensitivity and response kinetics in all Gγ-expressing lines

**Fig 6:**
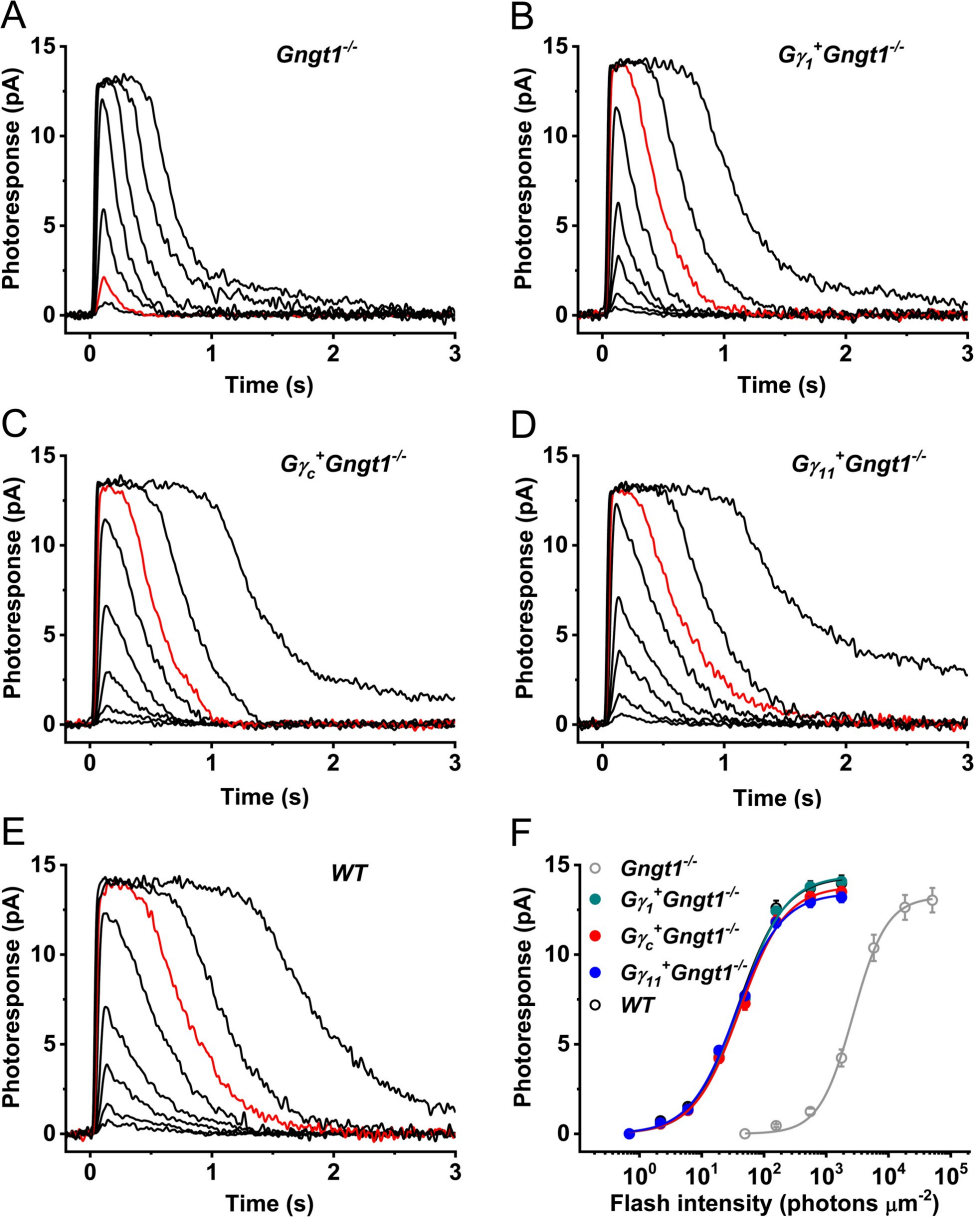
Light responses of rods in control and transgenic mouse lines expressing different Gγ-subunits. (**A–E**) Representative families of flash responses from 4–5-week-old control *Gngt1*^-/-^ (**A**), *Gγ*_*1*_^*+*^*Gngt1*^*-/-*^ (**B**), *Gγ*_*c*_^*+*^*Gngt1*^*-/-*^ (**C**), *Gγ*_*11*_^*+*^*Gngt1*^*-/-*^ (**D**), and wild type (**E**) mouse rods. Test flashes of 500 nm light with intensities of 160, 560, 1.8x10^3^, 5.8x10^3^, 1.8x10^4^, 5.1x10^4^, and 1.6x10^5^ photons μm^-2^ (for *Gngt1*^*-/-*^ rods) or 2, 6, 19, 50, 160, 560, 1.8x10^3^, and 5.8x10^3^ photons μm^-2^ (for wild type and all transgenic rods) were delivered at time 0. Red traces show responses to identical light intensity (560 photons μm^-2^). (**F**) Averaged intensity-response relationships (mean ± SEM) for *Gngt1*^-/-^ (n = 11), *Gγ*_*1*_^*+*^*Gngt1*^*-/-*^ (n = 31), *Gγ*_*c*_^*+*^*Gngt1*^*-/-*^ (n = 30), *Gγ*_*11*_^*+*^*Gngt1*^*-/-*^ (n = 24), and wild type (n = 8) mouse rods. Data were fitted with hyperbolic Naka-Rushton functions that yielded half-saturating light intensities (*I*_1/2_) indicated in Table 1. Error bars are smaller than the symbol size for most data points.

Next, we analyzed by suction electrode recordings whether the transgenic expression of the three different Gγ-subunits in individual *Gngt1*^*-/-*^ mouse rods would restore their photosensitivity and response kinetics. In agreement with the similar length of their outer segments at the age of 4–5 weeks (Fig 2) and normal ERG responses *in vivo* (Fig 5), *Gγ*_*1*_^*+*^*Gngt1*^*-/-*^, *Gγ*_*c*_^*+*^*Gngt1*^*-/-*^, and *Gγ*_*11*_^*+*^*Gngt1*^*-/-*^ rods produced saturated responses of similar amplitudes, not different from these in wild type and *Gngt1*^*-/-*^ cells (Fig 6A–6F and Table 1). Remarkably, compared to the dramatically desensitized (~70-fold) Gγ_1_-deficient rods, the light sensitivity of all transgenic photoreceptors was restored to wild type levels (Fig 6F). It should be noted, however, that the average sensitivity of *Gγ*_*11*_^*+*^*Gngt1*^*-/-*^ rods was slightly (~20%) higher than that in the other two Gγ-expressing lines (Table 1).

**Table 1:** Parameters of single-cell responses from dark-adapted mouse rods.

Response parameter	*Gngt1*^*-/-*^ (n = 11)	*Gγ*_*1*_^*+*^*Gngt1*^*-/-*^ (n = 31)	*Gγ*_*c*_^*+*^*Gngt1*^*-/-*^ (n = 30)	*Gγ*_*11*_^*+*^*Gngt1*^*-/-*^ (n = 24)	*WT* (n = 8)
*R*_max_ (pA)	13.2 ± 0.6 NS	14.1 ± 0.3 NS	13.5 ± 0.3 NS	13.2 ± 0.3 NS	14.0 ± 0.4
*T*_peak_ (ms)	108 ± 6 ***	153 ± 4 NS	162 ± 5 NS	152 ± 3 NS	157 ± 5
*T*_integr._ (ms)	177 ± 18 ***	286 ± 17 NS	290 ± 15 NS	278 ± 17 NS	297 ± 19
*S*_f_ (μm^2^ ph^-1^)	1.7x10^-4^ ± 2.0x10^-5^ ***	1.6x10^-2^ ± 8.9x10^-4^ NS	1.6x10^-2^ ± 1.1x10^-3^ NS	1.7x10^-2^ ± 1.2x10^-3^ NS	1.7x10^-2^ ± 1.0x10^-3^
*I*_1/2_ (ph μm^-2^)	3007 ± 308 ***	45 ± 2 NS	46 ± 3 NS	38 ± 2 NS	40 ± 3
*τ*_rec_ (ms)	146 ± 13 **	223 ± 16 NS	214 ± 14 NS	226 ± 16 NS	236 ± 13
*τ*_D_ (ms)	162 ± 15 ***	207 ± 11 **	240 ± 15 *	301 ± 16 NS	324 ± 17

*R*_max_, maximal dark current measured from saturated responses; time-to-peak (*T*_peak_), integration time (*T*_integr._), and normalized flash sensitivity (*S*_f_) refer to responses whose amplitudes were ∼0.2∙*R*_max_ and fell within the linear range; *I*_1/2_, half-saturating light intensity; *τ*_rec_, time constant of single-exponential decay of the dim flash response recovery phase; *τ*_D_, dominant time constant of recovery after supersaturating flashes determined from the linear fit to time in saturation vs. intensity semilog (Pepperberg) plots [23]. Data are presented as mean ± SEM. Student’s t-test, NS (not significant) indicates *p* > 0.05

* indicates *p* < 0.05

** indicates *p* < 0.01

*** indicates *p* < 0.001, all compared to wild type values.

**Fig 7:**
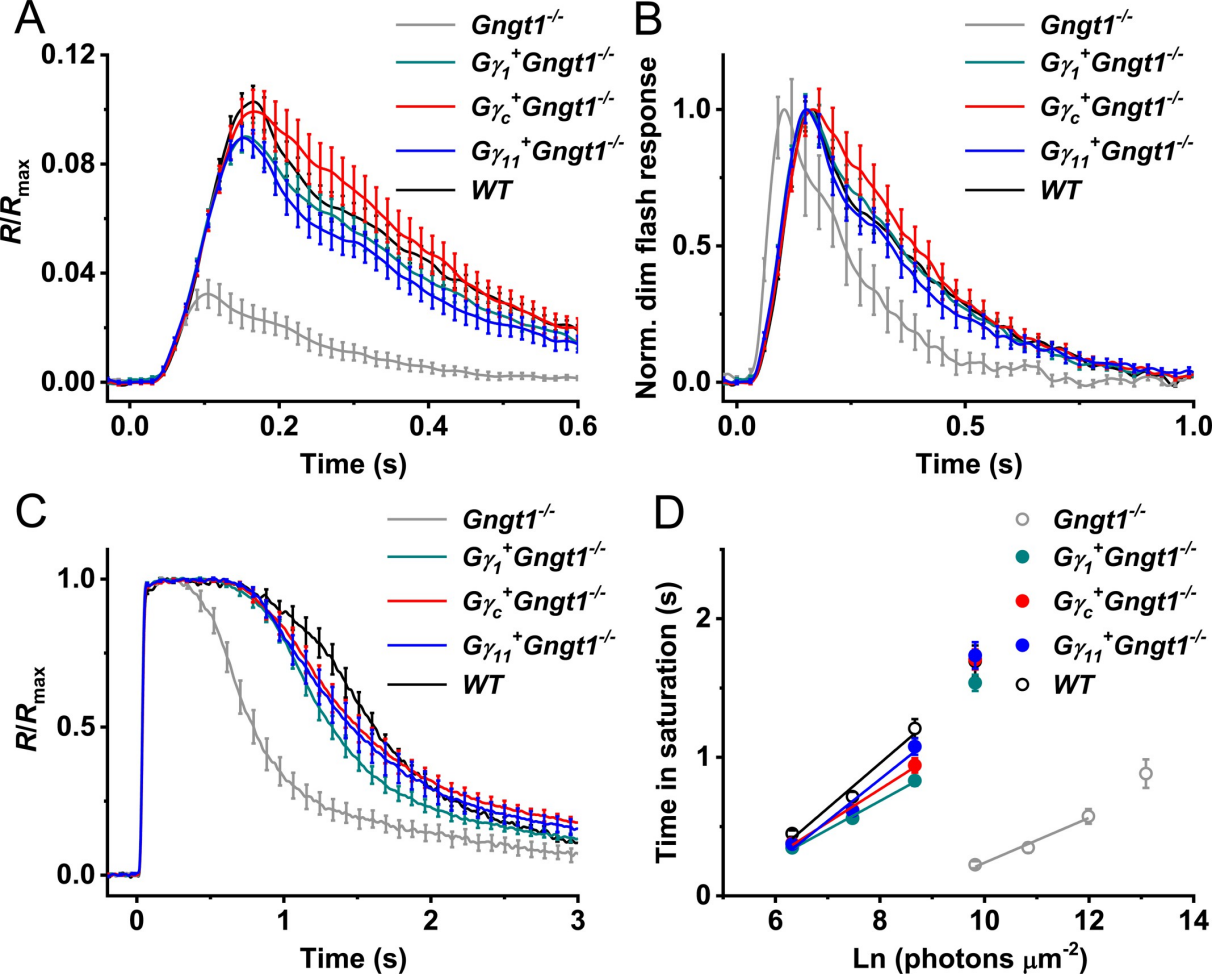
Activation and inactivation of rod phototransduction cascade in control and transgenic mice expressing different Gγ subunits. (**A**) Amplification of phototransduction in mouse rods. Dim flash responses (to light intensities of 560 photons μm^-2^ for *Gngt1*^-/-^ rods and 6 photons μm^-2^ for wild type and all transgenic Gγ-expressing rods) were normalized to maximum dark currents (*R*_max_) of the respective cells and population-averaged (mean ± SEM). Then, the *Gngt1*^-/-^, *Gγ*_*c*_^*+*^*Gngt1*^*-/-*^, and *Gγ*_*11*_^*+*^*Gngt1*^*-/-*^ responses were scaled to make their initial rising phase to coincide with that of the wild type response. Correspondingly scaled light intensities were 0.03:1:0.9:1.1:1 (*Gngt1*^*-/-*^:*Gγ*_*1*_^*+*^*Gngt1*^-/-^:*Gγ*_*c*_^*+*^*Gngt1*^*-/-*^:*Gγ*_*11*_^*+*^*Gngt1*^*-/-*^:*WT*), indicating ~30-fold higher gain in the Gγ-expressing rods. (**B**) Phototransduction shutoff in mouse rods. Dim flash responses (to light intensities of 560 photons μm^-2^ for control *Gngt1*^-/-^ rods and 6 photons μm^-2^ for wild type and all Gγ-expressing rods) were normalized to their own maximums and population-averaged (mean ± SEM). (**C**) Supersaturated responses (to light intensities of 1.6x10^5^ photons μm^-2^ for control *Gngt1*^-/-^ rods and 5.8x10^3^ photons μm^-2^ for wild type and all Gγ-expressing rods) were normalized to their amplitudes (*R*_max_) and population-averaged (mean ± SEM). (**D**) Determination of the dominant recovery time constant (*τ*_D_) from a series of supersaturating flashes for *Gngt1*^-/-^ (n = 11), *Gγ*_*1*_^*+*^*Gngt1*^*-/-*^ (n = 31), *Gγ*_*c*_^*+*^*Gngt1*^*-/-*^ (n = 30), *Gγ*_*11*_^*+*^*Gngt1*^*-/-*^ (n = 23), and wild type (n = 8) mouse rods. Linear fits yielded *τ*_D_-values indicated in Table 1. Values are means ± SEM (smaller than the symbol size for some data points).

We then evaluated the kinetics of activation of the rod phototransduction cascade in all three mutant mouse strains by directly comparing the light intensities required to produce identical initial phases of response activation (Fig 7A). In accordance with their restored sensitivity, the phototransduction amplification in *Gγ*_*1*_^*+*^*Gngt1*^*-/-*^ rods was increased by ~34-fold compared to that in cells lacking Gγ_1_ and reached wild type level, as evident from the analysis of rising phases of their dim flash responses during the first 40 ms after the test flash. The cascade activation was only slightly (~10%) lower in *Gγ*_*c*_^*+*^*Gngt1*^*-/-*^ rods and higher (by ~10%) in *Gγ*_*11*_^*+*^*Gngt1*^*-/-*^ cells than in the Gγ_1_-expressing transgenic rods, thus showing a comparable degree of restoration in all three transgenic lines.

One characteristic feature of *Gngt1*^*-/-*^ rods is the significantly faster inactivation of their signaling cascade, an effect contributing to their reduced photosensitivity [2]. In contrast, normal inactivation rate of dim flash responses was achieved in the rods of all transgenic lines expressing a Gγ-subunit, as judged from their normal time-to-peak, integration time, and single-exponential dim flash response recovery time constant (*τ*_rec_) (Fig 7B and Table 1). Coincidentally, the response recovery following supersaturating flashes was also slower in all transgenic lines than in Gγ_1_-deficient controls, as evident from comparing the kinetics of their maximal rod responses (Fig 7C) and the corresponding dominant recovery time constants (*τ*_D_) (Fig 7D and Table 1). All these parameters were also comparable to those typically observed in wild type mouse rods (Table 1 and [2]). It should be mentioned that the rods expressing Gγ_11_ had the slowest *τ*_D_ among all transgenic cells (Table 1) although the molecular mechanisms behind their slight response deceleration remain unclear. Taken together, these results indicate that the transgenic expression of various G-protein γ-subunits with distinct amino acid sequences rescues equally well the expression level of rod transducin α-subunit in Gγ_1_-deficient mouse rods and effectively restores their signaling, although with slightly different photoresponse kinetics.

### Light-driven translocation of Gtα_1_ in Gγ-expressing rods

**Fig 8:**
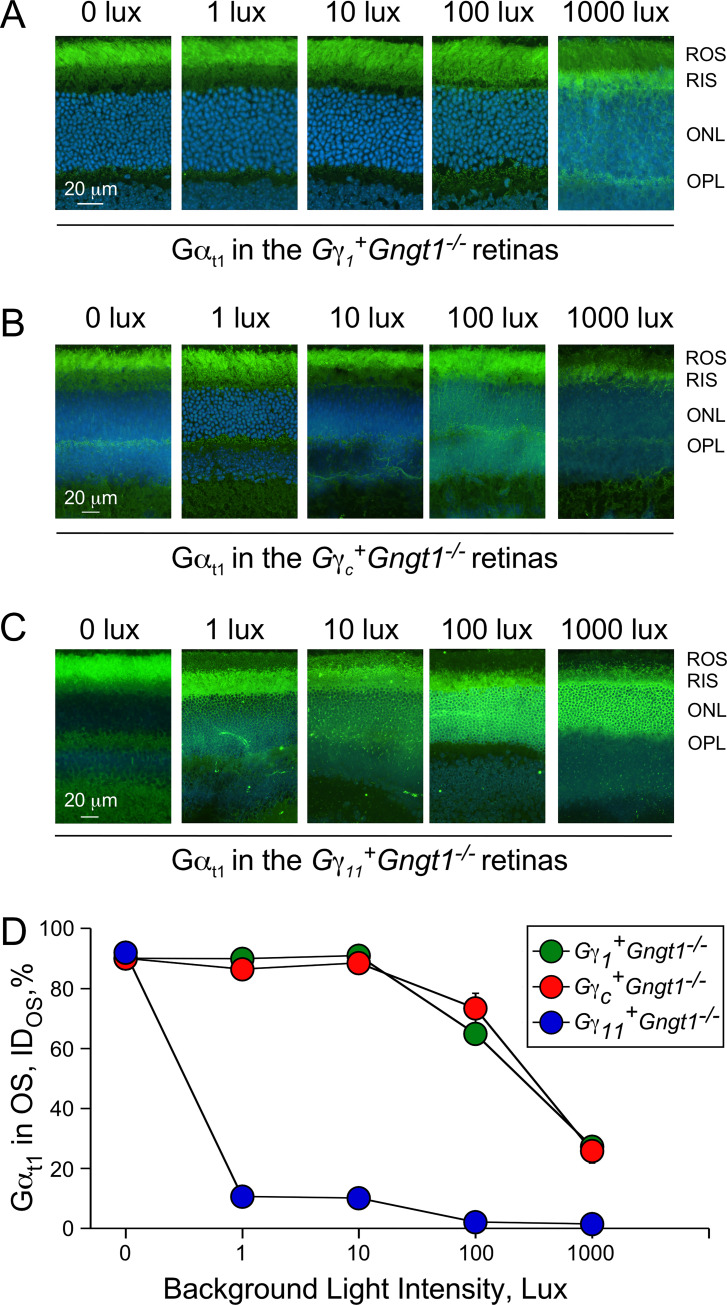
Translocation of Gα_t1_-subunit in Gγ transgenic retinas under various background light conditions. Immunohistochemical analysis of retinas stained with anti-Gα_t1_ antibody (green) and counterstained with DAPI (blue). Gα_t1_ distribution in retinas from the *Gγ*_*1*_^*+*^*Gngt1*^*-/-*^ (**A**), *Gγ*_*c*_^*+*^*Gngt1*^*-/-*^ (**B**), and *Gγ*_*11*_^*+*^*Gngt1*^*-/-*^ retinas (**C**). ROS–rod outer segments, RIS–rod inner segments, ONL–outer nuclear layer, OPL–outer plexiform layer. (**D**) Proportion of Gα_t1_ in OS vs. IS+ONL+OPL, percent integrated density (n = 3).

Finally, we investigated how the expression of each of the three transgenic Gγ subunits in rods affects the light-driven translocation of Gα_t1_ from the outer segment to the inner segment of these photoreceptors. We examined the distribution of Gα_t1_ across the rods in 5 different background light conditions: darkness and at 1, 10, 100, and 1000 lux of steady background illumination. To allow translocation to occur, dark-adapted animals were exposed to the background light for 15 minutes, and then were rapidly euthanized and their eyes were dissected, cryo-preserved, sectioned, and stained with the Gα_t1_ antibody for immunohistochemical analysis of its distribution. Consistent with the localization of the transgenic Gγ_1_, Gγ_c_, and Gγ_11_ subunits to the outer segments of rods in dark-adapted retinas (Fig 3), we found that Gα_t1_ was also properly localized in the rod outer segments in darkness (0 lux; Fig 8A–8C, left panels, and 8D). In *Gγ*_*1*_^*+*^*Gngt1*^*-/-*^ and *Gγ*_*c*_^*+*^*Gngt1*^*-/-*^ mice, approximately 90% of Gtα_t1_ remained in the outer segments in dim background illumination of 1 and 10 lux, and eventually translocated to the inner segments when the retinas were illuminated with 100 and 1000 lux of light (Fig 8A and 8B, right two panels). This is qualitatively consistent with previous work showing that in wild type mouse rods the threshold for transducin translocation is near 4.6x10^3^ R* rod^-1^ s^-1^ [29], and indistinguishable from the Gtα_t1_ translocation in wild type and *Gngt1*^*+/-*^ retinas under identical conditions. The *Gngt1*^*+/-*^ control contains one *Gngt1*-wild type copy and one *Gngt1*^*-*^ copy and could be used as a closer genetic match for *Gγ*_*1*_^*+*^*Gngt1*^*-/-*^ containing one copy of the *Gngt1* transgene and two *Gngt1*^*-*^ copies. In contrast, translocation of Gα_t1_ in *Gγ*_*11*_^*+*^*Gngt1*^*-/-*^ retinas was triggered with illumination as low as 1 lux (Fig 8C and 8D, blue circles). At 1 lux, only 10% of Gα_t1_ remained in the outer segments of the *Gγ*_*11*_^*+*^*Gngt1*^*-/-*^ retinas compared to 90% for the other two Gγ transgenes in respective lines (Fig 8D). The highly robust Gα_t1_ staining in the outer nuclear layer that is evident at 100 and 1000 lux in the *Gγ*_*11*_^*+*^*Gngt1*^*-/-*^ retinas is typically observed in wild type and *Gngt1*^*+/-*^ controls only at background illumination levels above 1000 lux. Thus, surprisingly, despite the essentially identical functional properties of dark-adapted rods expressing the three transgenic Gγ subunits, translocation of transducin during continuous light exposure was initiated at substantially lower light intensity in transgenic Gγ_11_ rods compared to transgenic Gγ_1_ or Gγ_c_ cells.

**Fig 9:**
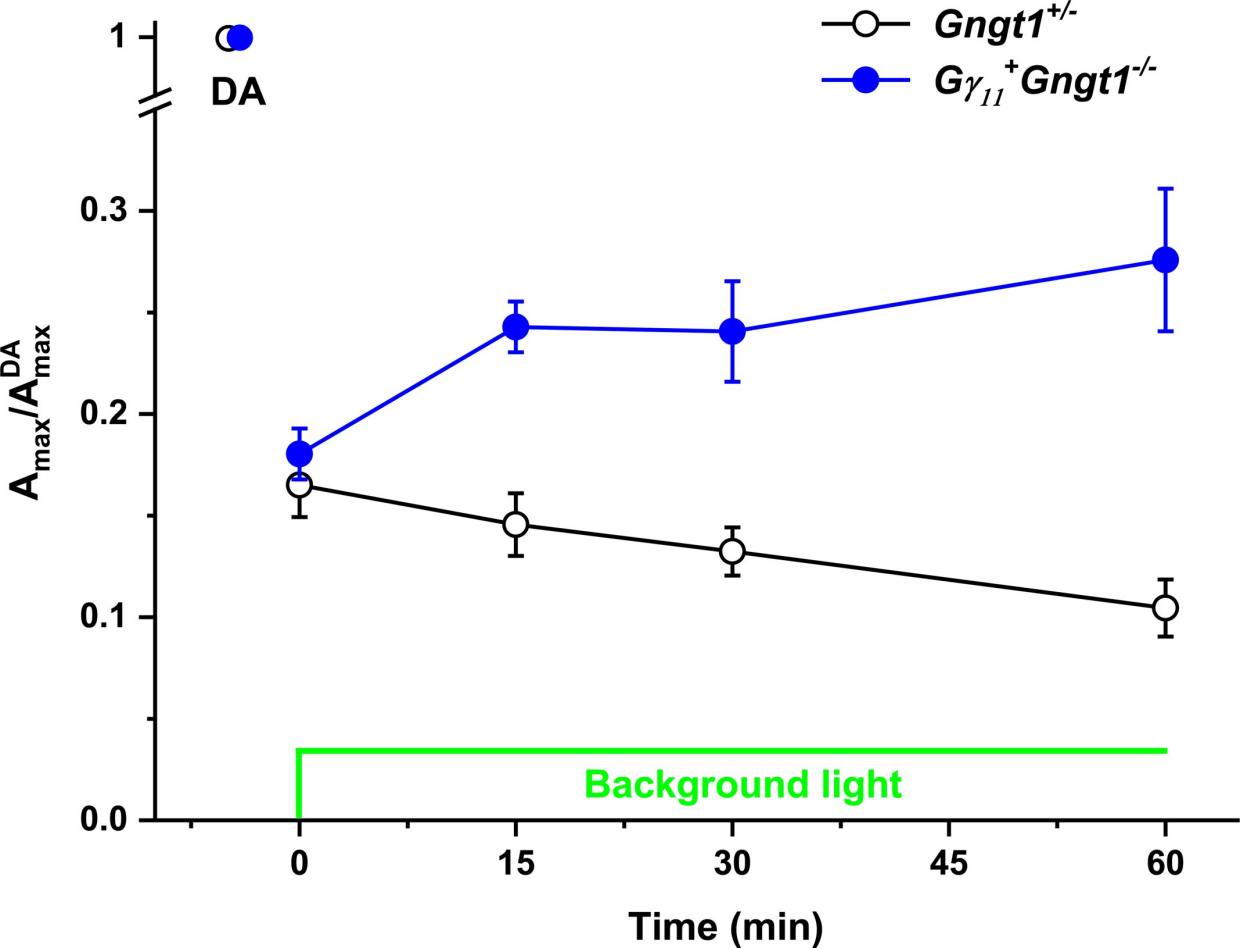
Changes of rod-driven maximal *ex vivo* transretinal ERG responses in control (*Gngt1*^*+/-*^) (n = 4) and transgenic *Gγ11+Gngt1*^*-/-*^ (n = 4) retinas. Retinas were exposed to prolonged moderate non-saturating 505-nm background light activating ~830 visual pigment molecules (R*) per rod per second initially. All maximal responses were normalized to corresponding dark-adapted response amplitudes (A^DA^_max_) and population-averaged. The onset and duration of background light are shown in green.

It was recently shown that the gradual translocation of transducin from the outer to the inner segments of rods under continuous illumination results in partial recovery of the rod response after its initial suppression by the background light [30]. Thus, we sought to determine whether the lower threshold for Gα_t1_ translocation found in *Gγ*_*11*_^*+*^*Gngt1*^*-/-*^ retinas affects the amplitude of the rod response over the course of 1-h exposure to background light. We used transretinal (*ex vivo* ERG) recordings to obtain and monitor the rod-driven responses. We exposed control *Gngt1*^*+/-*^ and transgenic *Gγ*_*11*_^*+*^*Gngt1*^*-/-*^ retinas to a moderate sub-saturating background light activating ~830 visual pigment molecules (R*) per rod per second at onset. This light would be expected to trigger transducin translocation in Gγ_11_ transgenic retinas but not in control retinas (Fig 8, see also [29]). As expected, in control retinas, the onset of the background light caused a rapid partial suppression of the rod maximal response (Fig 9, black symbols), which then persisted largely unchanged for the 60-min duration of the experiment, only slightly affected by a gradual rundown. The onset of an identical background light in *Gγ*_*11*_^*+*^*Gngt1*^*-/-*^ retinas produced comparable initial suppression of the rod maximal response. However, in stark contrast to the control case, the rod response then gradually recovered over the course of the 60 min of the experiment (Fig 9, blue symbols). As recently argued, this gradual increase reflects the translocation of Gα_t1_ away from the rod outer segments, which would effectively reduce the activation of the rod phototransduction by the steady background light, allowing the rods to recover partially their dark current [30]. Thus, the gradual recovery of rod responses in transgenic *Gγ*_*11*_^*+*^*Gngt1*^*-/-*^ retinas but not in control retinas in moderate background light is consistent with our observation that in these conditions transducin translocation takes place only in the transgenic *Gγ*_*11*_^*+*^*Gngt1*^*-/-*^ rods but not in controls (Fig 8).

## Discussion

Heterotrimeric G-proteins are the main transducers and amplifiers of extracellular signals from GPCRs to the intracellular effectors. It is now firmly established that specificity of the GPCR signaling and fine-tuning of the resulting physiological responses are regulated by the diversity of the Gα subunits, comprised of sixteen family members subdivided into four sub-families (G_s_, G_i/o_, G_q/11_, and G_12/13_), as well as by multiple combinations of five Gβ (Gβ_1–5_) and twelve Gγ (Gγ_1–13_) subunits. In many cell types containing various G-protein combinations, their interplay contributes to the rich gamut of cellular responses with defined spatio-temporal characteristics.

Retinal rod and cone photoreceptors provide a fascinating example of highly specialized sensory neurons that, while employing similar signaling architecture, differ drastically in their light sensitivity, photoresponse kinetics, and light adaptation properties. Being on the other side of the spectrum from a typical cell that contains multiple G-protein types, rods and cones rely on conserved cell-specific G-protein heterotrimers: Gα_t1_/Gβ_1_γ_1_ and Gα_t2_/Gβ_3_γ_c_, respectively [31]. While trace expression levels of Gγ_2_ and Gγ_3_ subunits were detected in rods, their physiological contribution in phototransduction is negligible [32]. This property makes rods a unique model system to study the physiological roles of G-protein subunits in visual transduction by substituting individual rod-specific G-protein subunits with their cone-specific or ubiquitous isoforms. This experimental design was successful to show that when Gα_t1_ was replaced by Gα_t2_ in rods, while retaining native rod Gβ_1_γ_1_ complex, the phototransduction was largely unaffected [5–7].

To determine the physiological role of Gβγ in photoreceptor function, we previously genetically removed the gene *Gngt1* encoding rod Gγ_1_ subunit and demonstrated that the high light sensitivity of rods and their robust signal amplification are severely compromised in mice [2]. The *Gngt1*^*-/-*^ model provided an excellent starting point to pose the next question of the possible physiological difference between various Gγ isoforms. Specifically, what is the reason for the selective use of Gγ_1_ and Gγ_c_ in rods and cones, respectively, and the exclusion of otherwise ubiquitously expressed Gγ_11_ from both photoreceptor types? This question is especially intriguing considering the fact that these three Gγ proteins belong to the same Class I Gγ subunits that are post-translationally modified by the shorter isoprenoid lipid farnesyl, as opposed to class II-IV Gγ subunits that are geranylgeranylated [33]. Farnesylation is required for proper targeting of G-proteins to the outer segment and full biological activity [34, 35]. Thus, replacing native rod Gγ_1_ with cone Gγ_c_ or Gγ_11_ subunit ensures highly controlled experimental conditions not affected by the Gγ class or isoprenylation differences.

Here, we generated three individual transgenic mouse lines expressing Gγ_c_, Gγ_11_, and control Gγ_1_ on the *Gngt1*^*-/-*^ background (Fig 2). Immunohistochemical staining of retina cross-sections for the FLAG epitope that was included in all transgenic constructs showed similarly healthy retina morphology, uniform expression of these Gγ proteins and their proper targeting to the rod outer segments (Fig 3). The levels of expression of other major phototransduction proteins, such as rhodopsin, transducin subunits, and PDE were identical between the experimental and control retinas (Fig 4). Transgenic re-introduction of Gγ_1_, Gγ_c_, or Gγ_11_ also completely restored the levels of endogenous Gα_t1_ (Fig 4) that is known to be severely reduced by the deletion of native Gγ_1_ [2, 3]. This result is of particular importance because signal amplification in mammalian rods is directly proportional to the level of expression of their Gα_t1_ subunit [27]. Thus, morphological and protein expression data argue that rods from the Gγ_1_, Gγ_c_, and Gγ_11_ transgenic lines are indistinguishable in their structure and protein complement.

Because Gβγ complexes function natively as inseparable heterodimers, the deletion of Gγ_1_ in rods is expected to lead to accumulation of misfolded Gβ_1_ protein. Slow progressive retinal degeneration in the Gγ_1_ deficient mice was proposed to be the result of proteostatic stress, or inability of the rod cell ubiquitin-proteasome system to degrade un-complexed Gβ_1_ protein effectively [36–39]. Expression of Gγ_1_, Gγ_c_, and Gγ_11_ in the Gγ_1_ deficient mice appears to rescue the retina degeneration phenotype independent of the type of the Gγ subunit, which argues for the productive complex formation of Gβ_1_γ_1_, Gβ_1_γ_c_, and Gβ_1_γ_11_ dimers and confirms previous biochemical results [40]. In addition, equal levels of the Gα_t1_ expression in transgenic retinas (Fig 4) and effective delivery of Gα_t1_ to the rod outer segments under dark adapted conditions (Fig 8) are consistent with normal heterotrimer formation and its proper subcellular localization.

There is a growing body of evidence that Gβγ-complexes contribute to the complexity and diversity of GPCR-mediated signaling that is shaped by specificity and response kinetics of GPCR/G-protein interactions at the plasma membrane, via direct interactions with effector molecules, as well as by acting at distant sites such as intracellular organelles [40, 41]. Thus, we examined whether Class I Gγ_1_, Gγ_c_, and Gγ_11_ modified by posttranslational farnesylation (Fig 1) would restore scotopic visual function, and to what extent they would determine rod photosensitivity and response kinetics. This question is especially intriguing while comparing and contrasting rod Gγ_1_ and cone Gγ_c_, as retinal rods respond to light at significantly lower light levels compared to cones, and rod response kinetics are markedly slower [42]. The results from our *in vivo* ERG experiments and single-cell suction electrode recordings conclusively demonstrate that despite minor variations, all three Class I Gγ subunits can support essentially normal scotopic rod photoresponses (Figs 5–7). Thus, the differences in Gγ composition between rods and cones cannot explain their unique activation properties in dark-adapted conditions. This also implies that Gγ involvement in the activation properties of photoreceptors per se has unlikely contributed to the evolutionary selection of Gγ_1_ for rods, Gγ_c_ for cones, and Gγ_11_ for other tissues. The physiological features determining selective expression of Gγ_1_ and Gγ_c_ in rods and cones is still to be determined. Our results mirror a previous observation obtained by replacing rod Gα_t1_ by cone Gα_t2_ that these two Gα_t_ isoforms are functionally interchangeable [5]. Knowing that neither Gα_t2_ nor Gγ_c_ makes the rod cascade activation cone-like, it remains quite possible that unique properties of cone phototransduction are determined by the Gγ_c_ counterpart Gβ_3_ as part of the unique cone Gβ_3_γ_c_ complex, as deletion of Gβ_3_ alone in cones doesn’t affect cone response kinetics [43]. Alternatively, differences in upstream and downstream phototransduction components [44–46], as well as structural differences between rods and cones could account for their unique functional characteristics.

In stark contrast to the functional interchangeability of Gγ_1_, Gγ_c_, and Gγ_11_ in dark-adapted rod phototransduction, we observed a significant effect by the Gγ composition on the cell responsiveness in steady background light. Upon increasing the intensity of background illumination rod responses saturate quickly, the process accompanied by massive light-driven translocation of Gα_t1_ from the rod outer to the rod inner segment [27]. While Gα_t1_ translocation was similar in Gγ_1_ and Gγ_c_ transgenic retinas, substitution of Gγ_1_ with Gγ_11_ shifted the light threshold that triggers translocation to lower background light intensity by 2–3 orders of magnitude (Fig 8). We observed that transducin in Gγ_11_ transgenic rods began to translocate at a light intensity of just 1 Lux, while Gγ_1_ and Gγ_c_ transgenic rods were still deeply dark-adapted. This remarkable effect had profound implications on rod function, as only Gγ_11_ transgenic rods recovered their response amplitudes under a moderate steady background light, as observed in our transretinal ERG recordings (Fig 9).

While Gγ_11_ is normally excluded from rods and cones [15], and thus transducin heterotrimer Gα_t1_Gβ_1_γ_11_ is likely not physiologically relevant, our results clearly demonstrate that in principle, the type of Gγ isoform can have significant implications for light adaptation and the kinetics of photoreceptors’ escape from physiological saturation. Because Gγ_1_, Gγ_c_, and Gγ_11_ belong to the same class of farnesylated Gγ subunits, the observed effect must be attributed to the unique amino acid sequence of Gγ_11_ (Fig 1). Interestingly, a previous study utilizing the knock-in of the geranylgeranylated mutant of Gγ_1_ demonstrated normal photoresponses but impaired photoresponse recovery caused by the stronger interaction of the mutant protein with lipid membranes and compromised light-driven translocation of Gt [47], a predictably opposite effect to what we observed with Gγ_11_. Similarly, a recent study with mutant Gα_t1_ that associates more strongly with Gβ_1_γ_1_ and as a result does not translocate efficiently in comparable background light, showed a suppressed recovery of the rod dark current under those conditions [30]. In the context of these findings, our results suggest that Gα_t1_ associates more weekly with Gβ_1_γ_11_ than with the endogenous Gβ_1_γ_1_, causing easier dissociation and translocation upon light exposure. This conclusion is also supported by the comprehensive biochemical analysis of the heterotrimeric G-protein complex formation that demonstrated significantly weaker association of Gβ_1_γ_11_ compared to Gβ_1_γ_1_ with Gα_i1_, a close relative of Gα_t1_ [48]. Taken together, it appears that the Gγ-subunit amino acid sequence and the prenylation identity contribute to the unique physiological properties of rod photoreceptors under continuous illumination.

## Conclusion

By replacing the native Gγ_1_ subunit in mouse rod photoreceptors with cone-specific Gγ_c_ or ubiquitous Gγ_11_ isoforms_,_ we examined the contribution of Gγ to the unique physiological properties of rods. Our results unequivocally show that while Class I Gγ subunits are functionally interchangeable in rod phototransduction, they control the light threshold for transducin translocation and the physiological light adaptation properties of rods.

## Supporting information

S1 Raw images Annotated Western blot images. (TIF)
