# Supplementary figures and images for: Regulation of rod photoreceptor function by farnesylated G-protein γ-subunits
Source: PLoS One. 2022 Aug 8;17(8):e0272506. doi: 10.1371/journal.pone.0272506 (PMC9359561; doi:10.1371/journal.pone.0272506)

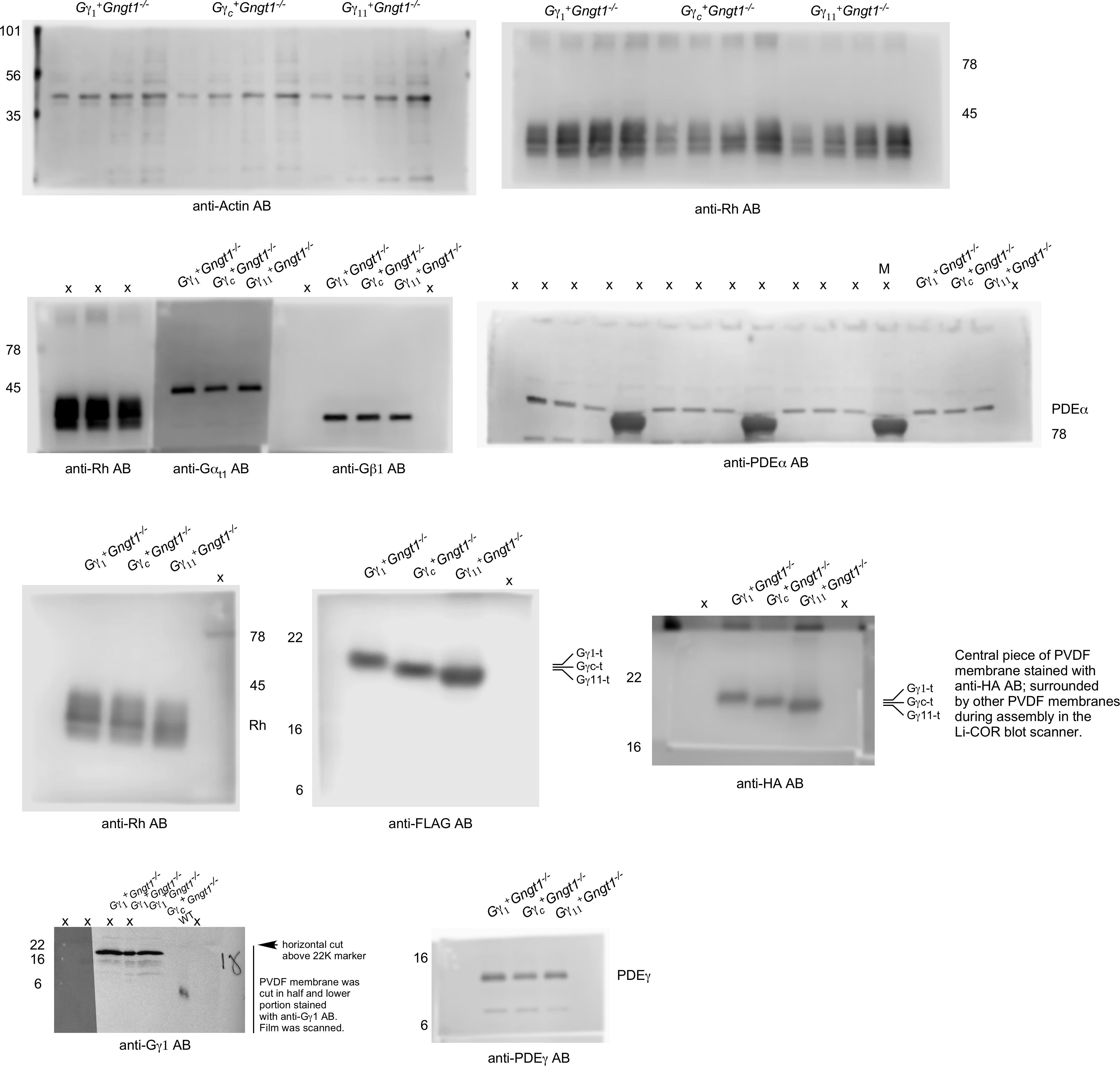

Supplement: S1 Raw images — (TIF) [file pone.0272506.s001.tif]
